# Supplementary material for: UAF1 deubiquitinase complexes facilitate NLRP3 inflammasome activation by promoting NLRP3 expression
Source: Nat Commun. 2020 Nov 27;11:6042. doi: 10.1038/s41467-020-19939-8 (PMC7695691; doi:10.1038/s41467-020-19939-8)
Supplement: Supplementary file 1 — Supplementary Information [file 41467_2020_19939_MOESM1_ESM.pdf]

Supplementary Information for

UAF1 deubiquitinase complexes facilitate NLRP3 inflammasome activation by promoting NLRP3 expression

Hui Song[1,2,4], Chunyuan Zhao[1,2,3,4], Zhongxia Yu[1,2], Qizhao Li[1,2], Rongzhen Yan[1,2], Ying Qin[1,2], Mutian Jia[1,2], and Wei Zhao[1,2,*]

[1] Department of Immunology, Key Laboratory for Experimental Teratology of the Chinese Ministry of Education, School of Basic Medical Science, Cheeloo College of Medicine, Shandong University, Jinan, Shandong 250012, China

[2] State Key Laboratory of Microbial Technology, Shandong University, Jinan, Shandong 250012, China

[3] Department of Cell Biology, School of Basic Medical Science, Cheeloo College of Medicine, Shandong University, Jinan, Shandong 250012, China

[4] These authors contributed equally to this work.

[*]Correspondence: wzhao@sdu.edu.cn (W.Z.)

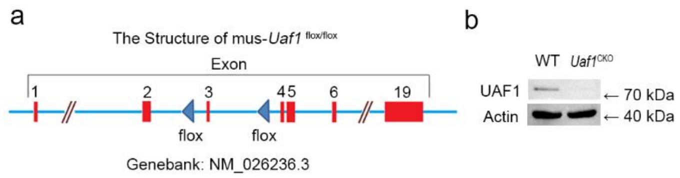

Supplementary Fig. 1 Efficiency of *Uaf1* knockout. **a** Generation of *Uaf1*$^{\text{flox/flox}}$ mice. Schematic illustration of the target region of *Uaf1*$^{\text{flox/flox}}$ mice. **b** Western blot analysis of UAF1 expression in mouse peritoneal macrophages obtained from WT or *Uaf1*^{CKO} mice. Similar results were obtained from three independent experiments.

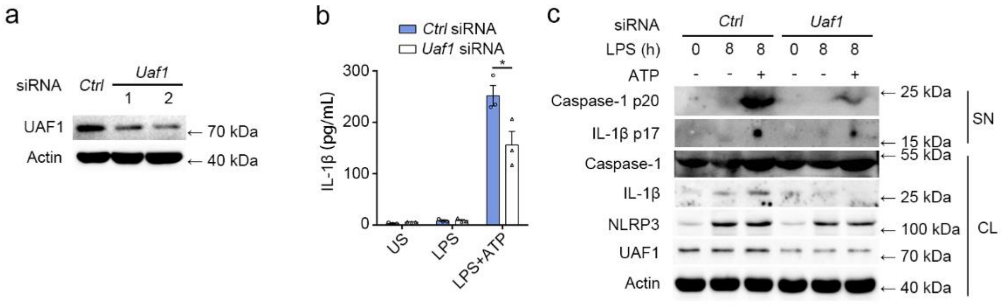

Supplementary Fig. 2 *Uaf1* knockdown inhibits NLRP3 inflammasome activation. a Western blot analysis of UAF1 expression in *Ctrl* siRNA-, *Uaf1* siRNA1- or *Uaf1* siRNA 2-transfected mouse peritoneal macrophages. **b, c** ELISA analysis of IL-1β secretion (**b**) and western blot analysis of caspase-1 and IL-1β cleavage (**c**) in *Ctrl* siRNA- or *Uaf1* siRNA-transfected mouse peritoneal macrophages followed by priming with LPS for 8 h and subsequent stimulation with ATP for 40 min (mean ± SEM, two-tailed t-test *Uaf1* siRNA vs *Ctrl* siRNA, *$P = 0.0441$; $n = 3$ independent experiments). US, unstimulated; SN, supernatants; CL, cell lysates. Similar results were obtained from three independent experiments.

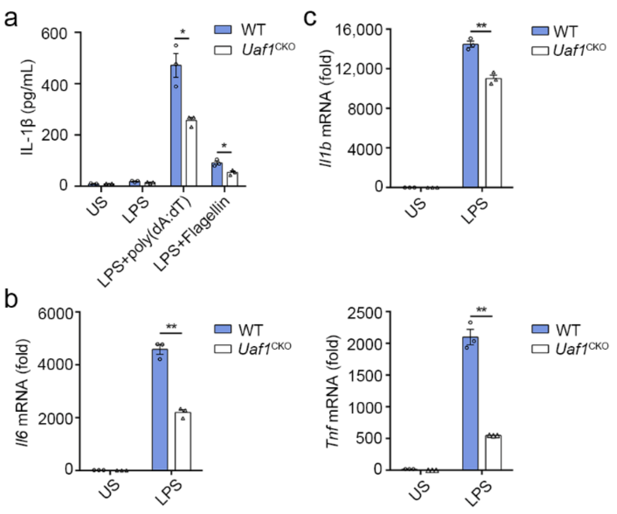

Supplementary Fig. 3 *Uaf1* deficiency inhibits mRNA expression of *Il1b*, *Il6*, and *Tnf*. a ELISA analysis of IL-1β secretion in mouse peritoneal macrophages from WT or *Uaf1*^{CKO} mice following priming with LPS for 8 h and subsequent stimulation with poly(dA:dT) or flagellin for 40 min (mean ± SEM, two-tailed t-test *Uaf1*^{CKO} vs WT, **P* = 0.0110, 0.0155 in sequence; *n* = 3 independent experiments). **b-c** RT-PCR analysis of *Tnf*, *Il6* **(b)** and *Il1b* **(c)** mRNA expression in mouse peritoneal macrophages from WT and *Uaf1*^{CKO} mice stimulated with LPS for 2 h (mean ± SEM, two-tailed t-test *Uaf1*^{CKO} vs WT, left panel: ***P* = 0.0004, right panel: ***P* =0.0002; *n* = 3 independent experiments in **(b)** and ***P* =0.0017; *n* = 3 independent experiments in **(c)**. US, unstimulated. Similar results were obtained from three independent experiments.

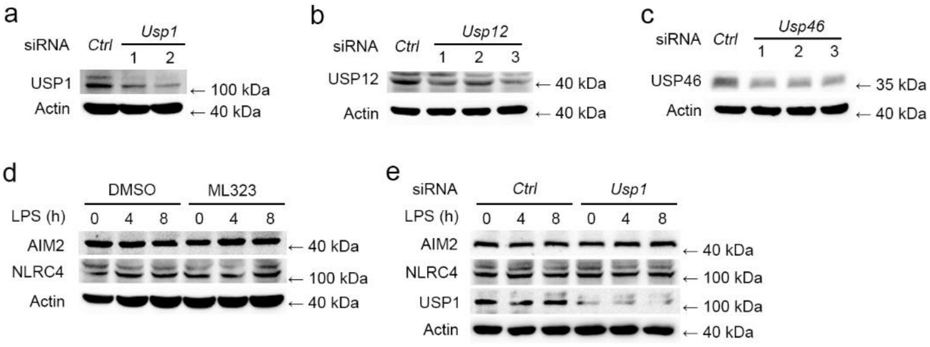

Supplementary Fig. 4 USP1 has no effect on the expression of AIM2 and NLRC4. a-c Western blot analysis of extracts of mouse peritoneal macrophages transfected with *Ctrl* siRNA, *Usp1* siRNA (**a**), *Usp12* siRNA (**b**), or *Usp46* siRNA (**c**). **d** Western blot analysis of AIM2 and NLRC4 expression in mouse peritoneal macrophages treated with DMSO or ML323, followed by stimulation with LPS for the indicated time periods. **e** Western blot analysis of AIM2 and NLRC4 expression in *Ctrl* siRNA- or *Usp1* siRNA 2-transfected mouse peritoneal macrophages, followed by stimulation with LPS for the indicated time periods. Similar results were obtained from three independent experiments.

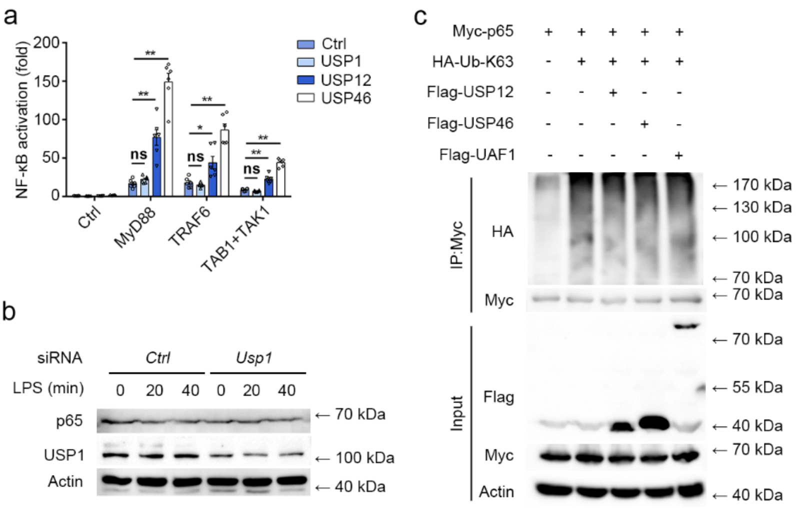

Supplementary Fig. 5 USP1 has no effect on NF-κB activation and p65 expression. a Luciferase analysis of NF-κB reporter activation in HEK293T cells transiently transfected with NF-κB reporter plasmid together with MyD88, TRAF6, or TAB1 and TAK1, and USP1, USP12, USP46 expression plasmids or empty control plasmid (mean ± SEM, two-tailed t-test USP1, USP12, USP46 plasmids vs empty control plasmid, ns=0902, ***P* = 0.0002, <0.0001, ns=0.3014, **P* =0.0131, ***P* <0.0001, ns=0.0561, ***P* =0.0001, <0.0001 in sequence; *n* = 6 independent experiments). **b** Western blot analysis of p65 in *Ctrl* siRNA- or *Usp1* siRNA-transfected mouse peritoneal macrophages, followed by stimulation with LPS for the indicated time periods. **c** Cell lysates of HEK293T cells transiently transfected with Myc-p65, HA-K63-Ub, Flag-USP12, Flag-USP46, and Flag-UAF1 were subjected to immunoprecipitation with Myc antibody, followed by western blot analysis with HA antibody. Similar results were obtained from three independent experiments.

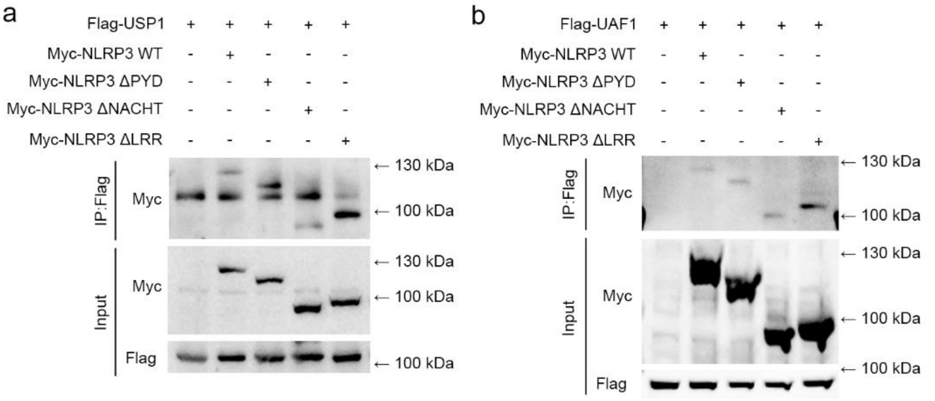

Supplementary Fig. 6 USP1 and UAF1 interacts with NLRP3 mutants. Myc-tagged NLRP3 or its mutants with Flag-USP1 (**a**) or Flag-UAF1 (**b**) were individually transfected into HEK293T cells. The cell lysates were immunoprecipitated with Flag antibody and then immunoblotted with the indicated antibodies. Similar results were obtained from three independent experiments.

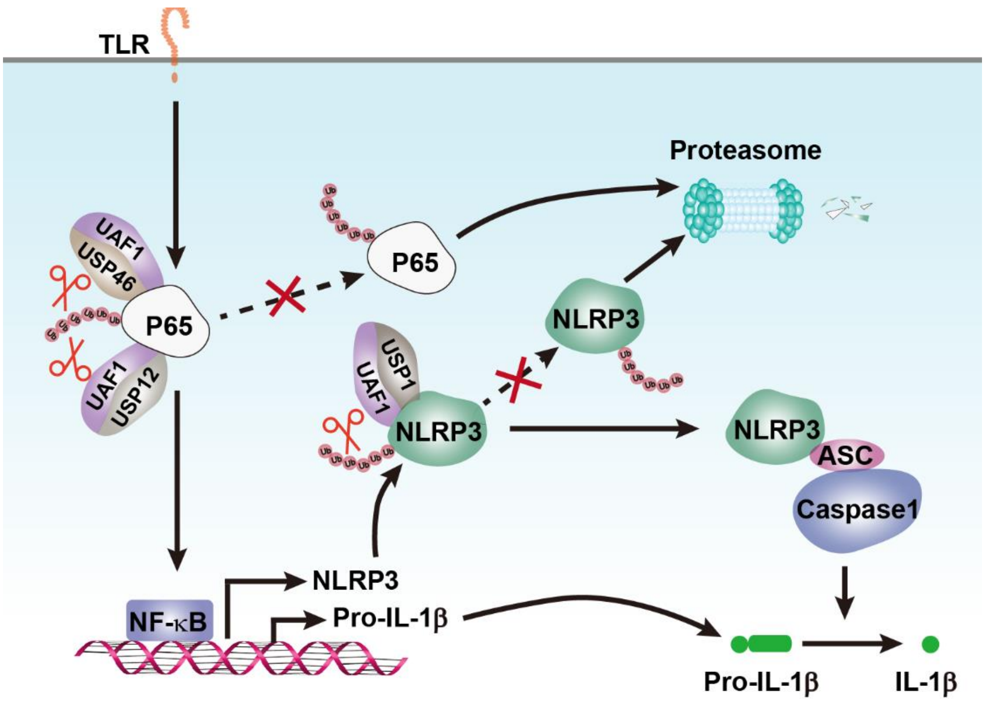

Supplementary Fig. 7 Work model for UAF1 facilitating NLRP3 inflammasome activation.

Supplementary Table 1. Sequences of PCR primers used in this study

Name	Prime	Sequence
mActb	Forward	5’-TGTTACCAACTGGGACGACA-3’
	Reverse	5’-CTGGGTCATCTTTTCACGGT- 3’
mNlrp3	Forward	5'-TGGATGGGTTTGCTGGGAT-3'
	Reverse	5'-CTGCGTGTAGCGACTGTTGAG-3'
mTnf	Forward	5'-GCCACCACGCTCTTCTGTCT-3'
	Reverse	5'-TGAGGGTCTGGGCCATAGAAC-3'
mIl6	Forward	5'-ACAACCACGGCCTTCCCTAC-3'
	Reverse	5'-CATTTCCACGATTTCCCAGA-3'
mIl1b	Forward	5'-ACCTTCCAGGATGAGGACATGA-3'
	Reverse	5'-AACGTCACACACCAGCAGGTTA-3'
