# Supplementary material for: UAF1 deubiquitinase complexes facilitate NLRP3 inflammasome activation by promoting NLRP3 expression
Source: Nat Commun. 2020 Nov 27;11:6042. doi: 10.1038/s41467-020-19939-8 (PMC7695691; doi:10.1038/s41467-020-19939-8)
Supplement: Supplementary file 3 — Reporting Summary [file 41467_2020_19939_MOESM3_ESM.pdf]

nature research

Corresponding author(s): Wei Zhao

Last updated by author(s): Oct 21, 2020

Reporting Summary

Nature Research wishes to improve the reproducibility of the work that we publish. This form provides structure for consistency and transparency in reporting. For further information on Nature Research policies, seeAuthors & Referees and theEditorial Policy Checklist .

Statistics

For all statistical analyses, confirm that the following items are present in the figure legend, table legend, main text, or Methods section.

n/a	Confirmed	
☐	☒	The exact sample size (*n*) for each experimental group/condition, given as a discrete number and unit of measurement
☐	☒	A statement on whether measurements were taken from distinct samples or whether the same sample was measured repeatedly
☐	☒	The statistical test(s) used AND whether they are one- or two-sided<br>*Only common tests should be described solely by name; describe more complex techniques in the Methods section.*
☒	☐	A description of all covariates tested
☒	☐	A description of any assumptions or corrections, such as tests of normality and adjustment for multiple comparisons
☐	☒	A full description of the statistical parameters including central tendency (e.g. means) or other basic estimates (e.g. regression coefficient) AND variation (e.g. standard deviation) or associated estimates of uncertainty (e.g. confidence intervals)
☐	☒	For null hypothesis testing, the test statistic (e.g. *F*, *t*, *r*) with confidence intervals, effect sizes, degrees of freedom and *P* value noted<br>*Give P values as exact values whenever suitable.*
☒	☐	For Bayesian analysis, information on the choice of priors and Markov chain Monte Carlo settings
☒	☐	For hierarchical and complex designs, identification of the appropriate level for tests and full reporting of outcomes
☒	☐	Estimates of effect sizes (e.g. Cohen's *d*, Pearson's *r*), indicating how they were calculated

Our web collection on statistics for biologists contains articles on many of the points above.

Software and code

Policy information about availability of computer code

Data collection	Applied Biosystems StepOnePlus (Thermo Fisher Scientific,USA), Amersham Imager 680 (GE Healthcare Life Sciences,USA), GeneGnome XR (Synoptics Ltd. Synoptics Ltd, England), Infinite M200 Pro (Tecan,Switzerland), LSM 780 with Airyscan (Zeiss, Germany).
Data analysis	ImageJ (ImageJ 1.38e, Bethesda, USA), Prism 6(GraphPad,California,USA).

For manuscripts utilizing custom algorithms or software that are central to the research but not yet described in published literature, software must be made available to editors/reviewers. We strongly encourage code deposition in a community repository (e.g. GitHub). See the Nature Research guidelines for submitting code & software for further information.

Data

Policy information about availability of data

All manuscripts must include a data availability statement. This statement should provide the following information, where applicable:

- Accession codes, unique identifiers, or web links for publicly available datasets
- A list of figures that have associated raw data
- A description of any restrictions on data availability

The authors declare that data that support this study are available within the article and its Supplementary Information files or available from the authors upon request.

Field-specific reporting

Please select the one below that is the best fit for your research. If you are not sure, read the appropriate sections before making your selection.

Life sciences study design

All studies must disclose on these points even when the disclosure is negative.

Sample size	Sample size for each experiment is indicated in the legend. No statistical methods were used to predetermine sample sizes. Sample size was chosen based on previous experiments and comparable. The reference of cellular experiments sample size is: H Chung, et al. NLRP3 regulates a non-canonical platform for caspase-8 activation during epithelial cell apoptosis. Cell Death & Differentiation. 23, pages1331–1346(2016). The reference of in vivo experiments is: Bolormaa Vandanmagsar et al. The NLRP3 inflammasome instigates obesity-induced inflammation and insulin resistance. Nature Medicine volume 17, pages179–188(2011).
Data exclusions	No data were excluded.
Replication	All experimental findings were reproduced in multiple independent experiments. For experiments using mouse peritoneal macrophages, each independent experiment used mouse peritoneal macrophages isolated from another mouse. For each figure, the number of independent experiments or biological replicates is indicated in the figure legends. Western blot pictures are from a representative experiment and the number of independent repeats is clearly indicated in the figure legends.
Randomization	No statistical methods were used for randomization. For in vitro experiments, mouse peritoneal macrophages were isolated from randomly chosen wild-type or CKO mice. For in vivo experiments, wild-type or CKO mice were randomly allocated into experimental groups.
Blinding	The investigators were blinded during data collection and analysis where possible, such as RT-PCR, ELISA and confocal microscopy.

Reporting for specific materials, systems and methods

We require information from authors about some types of materials, experimental systems and methods used in many studies. Here, indicate whether each material, system or method listed is relevant to your study. If you are not sure if a list item applies to your research, read the appropriate section before selecting a response.

Materials & experimental systems

n/a	Involved in the study	
☐	☒	Antibodies
☐	☒	Eukaryotic cell lines
☒	☐	Palaeontology
☐	☒	Animals and other organisms
☒	☐	Human research participants
☒	☐	Clinical data

Methods

n/a	Involved in the study	
☒	☐	ChIP-seq
☒	☐	Flow cytometry
☒	☐	MRI-based neuroimaging

Antibodies

Antibodies used	Anti-HA(HA-7, cat. H3663), anti-Myc(9E10, cat. M4439), and anti-Flag(M2, cat. F1804) were from Sigma-Aldrich.<br>Anti-NLRP3(Cryo-2, cat. AG-20B-0014), anti-Caspase-1 p20(Casper-1, cat. AG-20B-0042) were from AdipoGen.<br>Anti-USP46(polyclonal, cat. A17863) and anti-USP12(polyclonal, cat. A17862) were from ABclonal Technology.<br>Anti-Ub(P4D1, cat. sc-8017) and anti-β-actin(ACTBD11B7, cat. sc-8432) were from Santa Cruz Biotechnology.<br>Anti-Caspase-1(14F468, cat. GTX14367) was from GenTex.<br>Anti-p65(D14E12, cat. #8242), anti-p-p65(Ser536, cat. #3033), anti-IL-1β/p17(3A6, cat. #12242), anti-USP1(D37B4, cat. #8033) and anti-AIM2(polyclonal, cat. #13095) were from Cell Signaling Technology.<br>Anti-UAF1(polyclonal, cat. Ab122473) and anti-NLRC4(polyclonal, cat. Ab189593) were from Abcam.<br>Anti-Myc(9E10, cat. TA150121Z) and anti-HA(CB051, cat. TA180128-1) were from Origene.<br>Anti-NLRP3(polyclonal, cat. 19771-1-AP) was from Proteintech.<br>Anti-mouse secondary antibodies conjugated to Alexa Fluor 488(polyclonal, cat. A-11029) and anti-rabbit secondary antibodies conjugated to Alexa Fluor 633(polyclonal, cat. A-21070) were from Thermo Fisher Scientific.
Validation	All antibodies were obtained from indicated commercial vendors with ensured quality. All the antibodies used in this study have been validated by the vendors as indicated on the websites. Citations are listed as below:<br>Anti-HA(Sigma-Aldrich, cat. H3663) validate in human for WB: Toshiki Kamada et al. Cloning of Hynobius lichenatus (Tohoku hynobiid salamander) p53 and analysis of its expression in response to radiation. BMC Genetics. 21(1):53(2020).<br>Anti-Myc(Sigma-Aldrich, cat. M4439) validate in human for WB: Yi Yang et al. Cytoplasmic DAXX drives SQSTM1/p62 phase condensation to activate Nrf2-mediated stress response. Nature Communications.10(1):3759(2019).<br>Anti-Flag(Sigma-Aldrich, cat. F1804) validate in human for WB/IP: Huifang Hu et al. ZKSCAN3 counteracts cellular senescence by stabilizing heterochromatin. Nucleic Acids Research.48(11):6001-6018 (2020).<br>Anti-NLRP3(AdipoGen, cat. AG-20B-0014) M. Zheng, et al.Caspase-6 Is a Key Regulator of Innate Immunity, Inflammasome Activation, and Host Defense. Cell 181, 674 (2020).<br>Anti-Caspase-1 p20(AdipoGen, cat. AG-20B-0042) validate in mouse for WB: O. Gross, et al. Inflammasome Activators Induce Interleukin-1alpha Secretion via Distinct Pathways with Differential Requirement for the Protease Function of

Caspase-1.Immunity 36, 388 (2012).
Anti-USP46(ABclonal Technology, cat. A17863) and anti-USP12(ABclonal Technology, cat. A17862) were validate in multiple experiments to detect intended proteins in control samples with expected molecular weight to validate their effectiveness in our study .
Anti-Ub(Santa Cruz Biotechnology, cat. sc-8017) validate in mouse for WB: Benjamin P Woodall et al. Parkin does not prevent accelerated cardiac aging in mitochondrial DNA mutator mice. JCI Insight. 5(2019).
Anti-β-actin(Santa Cruz Biotechnology, cat. sc-8432) validate in human/mouse for WB: Rajeswari Jayavaradhan et al.CRISPR-Cas9 fusion to dominant-negative 53BP1 enhances HDR and inhibits NHEJ specifically at Cas9 target sites. Nature Communications. 10 (1):2866(2019), Mariafausta Fischietti et al. Slfn2 Regulates Type I Interferon Responses by Modulating the NF-κB Pathway. Molecular and Cellular Biology. 38(16)(2018).
Anti-Caspase-1(GenTex, cat. GTX14367) validate in human/mouse for WB: Subhash Haldar et al. Histone deacetylase inhibitors mediate DNA damage repair in ameliorating hemorrhagic cystitis. Scientific Reports. 6:39257(2016).
Anti-p65(Cell Signaling Technology, cat. #8242) validate in mouse for IP/WB: Josiane Fernandes Silva et al. Acute Increase in O-GlcNAc Improves Survival in Mice With LPS-Induced Systemic Inflammatory Response Syndrome.Frontiers in Physiology. 10 (1614) (2020).
Anti-p-p65(Cell Signaling Technology, cat. #3033) validate in mouse for WB: Insup Choi et al. Microglia clear neuron-released α-synuclein via selective autophagy and prevent neurodegeneration. Nature Communications.11(1):1386(2020).
Anti-IL-1β/p17(Cell Signaling Technology, cat. #12242) validate in mouse for WB: Alessandro Rimessi et al. Pharmacological modulation of mitochondrial calcium uniporter controls lung inflammation in cystic fibrosis. Science Advances. 6(19):eaax9093 (2020).
Anti-USP1(Cell Signaling Technology, cat. #8033) validate in human for WB: Benjamin B Shields et al. A genome-scale screen reveals context-dependent ovarian cancer sensitivity to miRNA overexpression. Molecular Systems Biology. 11(12):842(2015).
Anti-AIM2(Cell Signaling Technology, cat. #13095) validate in mouse for WB: Min Xie et al. PKM2-dependent glycolysis promotes NLRP3 and AIM2 inflammasome activation.Nature Communications. 7:13280(2016).
Anti-UAF1(Abcam, cat. Ab122473) validate in mouse for WB/IP: Zhongxia Yu et al. USP1-UAF1 deubiquitinase complex stabilizes TBK1 and enhances antiviral responses. The Journal of Experimental Medicine. 214(12):3553-3563(2017).
Anti-NLRC4(Abcam, cat. Ab189593) validate in mouse for WB: Guoji Zhu et al. Expression of NLRC4 in children with septicaemia and mechanisms of NLRC4 in in vitro cytokine secretion. Molecular medicine reports. 2016 Jul;14(1):509-14.
Anti-Myc(Origene, cat. TA150121) validate in human for IP: Li S et al. SHP2 Positively Regulates TGFß1-induced Epithelial-mesenchymal Transition Modulated by Its Novel Interacting Protein Hook1. J. Biol. Chem.289(49):34152-60(2014).
Anti-HA(Origene, cat. TA180128) validate in human for IP:Hu MM et al. Virus-induced accumulation of intracellular bile acids activates the TGR5-β-arrestin-SRC axis to enable innate antiviral immunity. Cell Research. 29(3): 193–205(2019).
Anti-NLRP3(Proteintech, cat. 19771-1-AP) validate in mouse for IP by using another NLRP3 antibody(AdipoGen, cat. AG-20B-0014) through WB.

Eukaryotic cell lines

Policy information about cell lines

Cell line source(s)	Human embryonic kidney (HEK293T) cells were obtained from American Type Culture Collection (Manassas, VA).
Authentication	None of the cell lines have been authenticated.
Mycoplasma contamination	The cell lines were not tested for mycoplasma contamination.
Commonly misidentified lines (See ICLAC register)	No commonly misidentified cell lines were used.

Animals and other organisms

Policy information about studies involving animals; ARRIVE guidelines recommended for reporting animal research

Laboratory animals	UAF1 flox/flox mice on a C57BL/6J background were generated by Cyagen Biosciences Inc.(Guangzhou, China). Lyz2Cre mice (stock number: 004781) were from Jackson Laboratory. UAF1flox/flox mice were crossed with Lyz2Cre mice to obtain UAF1-CKO mice with UAF1 deficiency in myeloid cells (UAF1-CKO). For animal experiments with UAF1-CKO mice, littermate controls with normal UAF1 expression (UAF1flox/flox) were used. C57BL/6 mice were from Vital River Laboratory Animal Technology Co. (Beijing, China). Mouse primary peritoneal macrophages were obtained from 4–6 weeks old female mice. Mouse embryonic fibroblasts were generated from 10 weeks female pregnant for 13-14 days mice. For in vivo experiments, 6 weeks old females were used.
Wild animals	Study did not involve wild animals.
Field-collected samples	Study did not involve samples collected from field.
Ethics oversight	All animal experiments were undertaken in accordance with the National Institute of Health Guide for the Care and Use of Laboratory Animals, with the approval of the Scientific Investigation Board of Medical School of Shandong University (Jinan, Shandong Province, China).

Note that full information on the approval of the study protocol must also be provided in the manuscript.
